# Neurodevelopmental Outcomes of Normocephalic Colombian Children with Antenatal Zika Virus Exposure at School Entry

**DOI:** 10.3390/pathogens13020170

**Published:** 2024-02-13

**Authors:** Sarah B. Mulkey, Elizabeth Corn, Meagan E. Williams, Colleen Peyton, Regan Andringa-Seed, Margarita Arroyave-Wessel, Gilbert Vezina, Dorothy I. Bulas, Robert H. Podolsky, Michael E. Msall, Carlos Cure

**Affiliations:** 1 Prenatal Pediatrics Institute, Children’s National Hospital, Washington, DC 20010, USA; elizabeth.corn@pennmedicine.upenn.edu (E.C.); mewilliams@childrensnational.org (M.E.W.); randringas@childrensnational.org (R.A.-S.); marroyave@childrensnational.org (M.A.-W.); 2 Department of Neurology, School of Medicine and Health Sciences, The George Washington University, Washington, DC 20037, USA; 3 Department of Pediatrics, School of Medicine and Health Sciences, The George Washington University, Washington, DC 20037, USA; 4 Department of Physical Therapy and Human Movement Sciences, Northwestern University Feinberg School of Medicine, Chicago, IL 60611, USA; colleen.peyton1@northwestern.edu; 5 Division of Radiology, Children’s National Hospital, Washington, DC 20010, USA; gvezina@childrensnational.org (G.V.); dbulas@childrensnational.org (D.I.B.); 6 Division of Biostatistics and Study Methodology, Children’s National Hospital, Washington, DC 20010, USA; rpodolsky@childrensnational.org; 7 Kennedy Research Center on Intellectual and Neurodevelopmental Disabilities, University of Chicago Medicine, Chicago, IL 60637, USA; mmsall@bsd.uchicago.edu; 8 BIOMELab, Atlántico, Barranquilla 080001, Colombia; cacurec@gmail.com

**Keywords:** infectious disease, child development, executive function, motor, COVID-19, Movement Assessment Battery for Children, Behavior Rating Inventory of Executive Function, telehealth, brain imaging, Latin America

## Abstract

The long-term neurodevelopmental effects of antenatal Zika virus (ZIKV) exposure in children without congenital Zika syndrome (CZS) remain unclear, as few children have been examined to the age of school entry level. A total of 51 Colombian children with antenatal ZIKV exposure without CZS and 70 unexposed controls were evaluated at 4–5 years of age using the Behavior Rating Inventory of Executive Function (BRIEF), the Pediatric Evaluation of Disability Inventory (PEDI-CAT), the Bracken School Readiness Assessment (BSRA), and the Movement Assessment Battery for Children (MABC). The mean ages at evaluation were 5.3 and 5.2 years for cases and controls, respectively. Elevated BRIEF scores in Shift and Emotional Control may suggest lower emotional regulation in cases. A greater number of cases were reported by parents to have behavior and mood problems. BSRA and PEDI-CAT activity scores were unexpectedly higher in cases, most likely related to the COVID-19 pandemic and a delayed school entry among the controls. Although PEDI-CAT mobility scores were lower in cases, there were no differences in motor scores on the MABC. Of 40 cases with neonatal neuroimaging, neurodevelopment in 17 with mild non-specific findings was no different from 23 cases with normal neuroimaging. Normocephalic children with ZIKV exposure have positive developmental trajectories at 4–5 years of age but differ from controls in measures of emotional regulation and adaptive mobility, necessitating continued follow-up.

## 1. Introduction

Although Zika virus (ZIKV) transmission to humans was documented as early as 1952, by far its largest epidemic occurred in Latin America from 2015 to 2016 [1]. Following the first reported case of ZIKV in Brazil in May 2015 and an alarming subsequent increase in the prevalence of birth defects in the region, countries in Latin America and across the world began to more broadly monitor this flavivirus [2]. In recognition of its teratogenic effects and rapid spread by Aedes mosquitos in the region, ZIKV was declared a public health emergency by the World Health Organization in February 2016 [2]. From August 2015 to April 2016, Colombia saw a total of 65,726 reported cases of Zika virus, 11,944 of which were diagnosed in pregnant women [3].

While symptoms of ZIKV infection are mild in most adults, the fetal developing brain can be severely impacted by in utero exposure to the virus. It is estimated that 5–13% of infants with antenatal ZIKV exposure present with microcephaly or other ZIKV-related birth defects including hearing loss, ophthalmological findings, or arthrogryposis, collectively termed congenital Zika syndrome (CZS) [4]. However, the majority of infants born after antenatal ZIKV exposure do not have CZS since they appear normal at birth and do not have apparent birth defects. Potential long-term effects of in utero ZIKV exposure in children without CZS thus remain unclear.

The US Centers for Disease Control and Prevention has recommended the long-term follow up of all ZIKV-exposed children due to potential neurodevelopmental effects [5], yet many longitudinal outcome studies which have assessed the risk of neurodevelopmental delays to date have only followed children until approximately two years of age [6]. Children born during the 2015–2016 Zika outbreaks in Latin America have now reached the age of school entry. Building on the findings of Mulkey et al., 2023 [7], this paper presents the longitudinal outcomes of a well-characterized Colombian cohort of ZIKV-exposed children without CZS at ages 4–5 years. These children have been seen for neurodevelopmental follow-up as infants and toddlers at approximately 6 months, 18 months, and 3 years of age, as part of an international collaboration between researchers in Barranquilla, Colombia and Washington, DC, USA beginning in 2016 [7,8]. As these children approach the early school-age years, we seek to examine whether there are neurodevelopmental differences in executive function, motor ability, language development, or scholastic skills as compared to a group of unexposed control participants from the same communities in Colombia. At the three year timepoint, ZIKV-exposed cases were found to have higher T-scores on the Behavior Rating Inventory of Executive Function—Preschool edition (BRIEF-P) than controls in shift and flexibility domains, indicating lower emotional regulation skills [7]. A higher percentage of parents of ZIKV-exposed cases reported mood problems than parents of controls [7]. Cases also had lower Pediatric Evaluation of Disability Inventory—Computer Adaptive Test (PEDI-CAT) T-scores in the mobility domains compared to controls [7].

The objective of this study was to assess the multi-domain neurodevelopmental outcomes in 4–5-year-old children with antenatal ZIKV exposure without CZS compared to unexposed controls in Colombia. While our prior study compared the same ZIKV-exposed cases and controls, the cases were one year younger [7]. In the present study, case and control child data are compared at the same age. We hypothesized that ZIKV-exposed cases would continue to show differences in emotional regulation compared to controls and may also display differences in other aspects of executive function, language development, and motor ability. As a secondary objective, we aimed to assess whether the neurodevelopmental outcomes of children with antenatal ZIKV exposure differ based on whether or not they had mild non-specific neuroimaging findings on neonatal imaging studies.

The study concludes that ZIKV exposure may impact child emotional regulation and adaptive mobility. The presence of mild non-specific neuroimaging findings was not associated with negative child neurodevelopment at age 4–5 years among cases.

## 2. Materials and Methods

### 2.1. Study Population

We performed a prospective case–control study of 51 ZIKV-exposed children (cases) and 70 unexposed children (controls) in Sabanalarga, Department of Atlántico, Colombia. All children lived in either the semi-rural town of Sabanalarga or in the rural towns of La Peña or Aguada de Pablo. Cases had laboratory-confirmed prenatal exposure to ZIKV during a period of endemicity in Colombia (2015–2017). The case cohort was followed serially since the prenatal period with pre and postnatal magnetic resonance imaging (MRI) exams, antenatal obstetric ultrasound, and postnatal cranial ultrasound exams, and had previous neurodevelopmental evaluations at eighteen months and three years of age [7,8,9]. All eligible cases had normal fetal imaging, were normocephalic at birth, and had no clinical findings of CZS at birth [9]. Children with postnatal neuroimaging had only normal or non-specific findings [9].

The 70 controls were prospectively enrolled as previously described in Mulkey et al., 2023 [7]. Controls were at least one year older than cases to preclude asymptomatic prenatal exposure to ZIKV during at least the first half of gestation; however, cases and controls were both assessed at age 4–5 years. Children were excluded as a control if their mother reported exposure to an infectious disease during pregnancy, such as Zika, Dengue, or Chikungunya or if the child was born preterm (≤36 weeks); had a chronic medical, behavioral, or psychological condition; received therapy for a developmental condition; had a history of seizures, abnormal hearing or vision not corrected by lenses; or had other developmental concerns expressed by family members or caregivers [7].

This study received approval from the Children’s National Hospital Institutional Review Board, Washington, DC and the Institutional Review Committee and Independent Committee on Research Ethics (CIRCIE), Barranquilla, Colombia. Women provided written informed consent for the participation of their child in the study. The study followed the Strengthening the Reporting of Observational Studies in Epidemiology (STROBE) reporting guidelines. This study is registered on ClinicalTrials.gov, NCT04398901.

### 2.2. Setting

**Figure 1:**
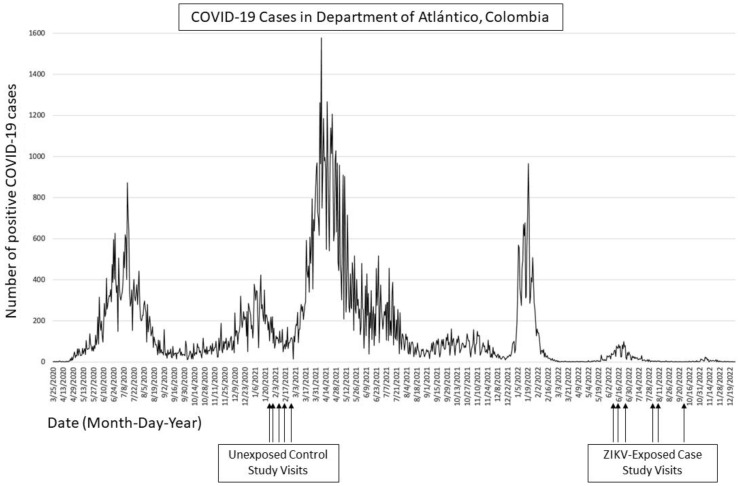
A timeline of the 4–5-year evaluations of ZIKV-exposed cases and unexposed controls relative to the progression of the COVID-19 pandemic in the Department of Atlántico, Colombia. Created using publicly available COVID-19 case data from the Colombian Instituto Nacional de Salud (National Institute of Health) [10].

**Figure 2:**
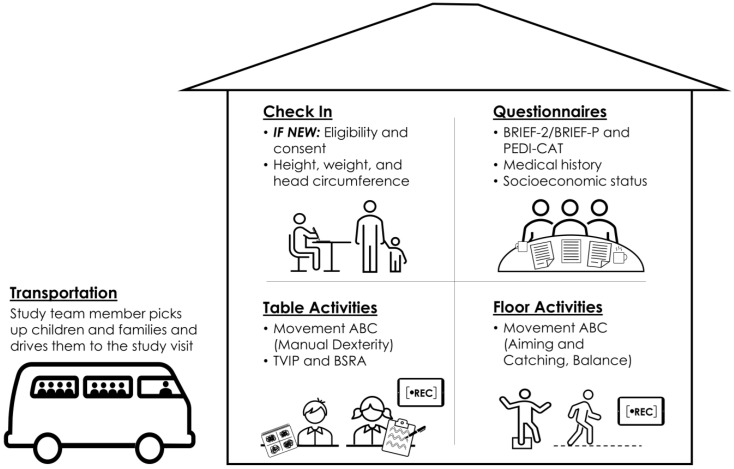
Organization of study visits using the ZIKV Outcome Toolbox (originally described in Mulkey et al., 2023 [7]). All study visits were completed in local community centers in Sabanalarga, Department of Atlántico, Colombia. Participants and adult family members completed multiple activity stations, each overseen by a trained study team member. Table and floor activities were recorded and monitored using telehealth Zoom by US team members.

Controls were previously recruited for the study by door-to-door visits with research staff in local towns of the Department of Atlántico, Colombia [7]. Due to the age differences between case and control cohorts, each group was evaluated at a different point in time. Controls were evaluated at age 4–5 between 28 January 2021 and 18 February 2021, and cases were evaluated at the same age range between 10 June 2022 and 23 September 2022. The timing of the evaluations of cases and controls was influenced by the progression of the COVID-19 pandemic in the Department of Atlántico, Colombia. Control study visits occurred during a low point in COVID-19 cases in late January and early February of 2021, while cases were seen once COVID-19 rates had dropped significantly in the summer and fall of 2022. (Figure 1). All evaluations were conducted at local multipurpose community centers rented for the study visits. Community centers were selected for available space for movement and seated activities and internet connectivity for livestreaming to team members in the USA (Figure 2). Telehealth Zoom was used to record all study visits for live monitoring by the US team. Video recordings were transferred to the US team via secure Dropbox folders to verify the proper scoring of activities.

### 2.3. ZIKV Outcome Toolbox

Neurodevelopment was assessed using the ZIKV Outcome Toolbox [7]. The toolbox was implemented for this study timepoint as depicted in Figure 2, with participants and their parents or caregivers completing activities across several stations inside the community center. The Spanish language version of the caregiver questionnaire Behavior Rating Inventory of Executive Function (BRIEF [PAR, Inc.]) was utilized to assess emerging executive function, including behavioral, emotional, and cognitive regulation [11]. Two different versions of the BRIEF were administered; controls completed the BRIEF for Preschool (BRIEF-P) and cases completed the BRIEF-2 [12,13]. The Spanish version of the Pediatric Evaluation of Disability Inventory—Computer Adaptive Test (PEDI-CAT, [Pearson]) was also completed by caregivers and administered by a trained study team member. The PEDI-CAT assesses ability in the domains of Daily Activities, Mobility, Social/Sognitive, and Responsibility [14]. Information on child medical history, parent education, and the home environment were also ascertained from caregivers. Mothers of ZIKV-exposed cases were also asked to share their experiences with Zika stigmatization and the effects on maternal mental health following their diagnosis during pregnancy and while parenting. While most caregivers independently completed questionnaires on paper, a trained research coordinator administered these items orally to parents or caregivers, when necessary, based on level of parent or caregiver literacy.

Two observational evaluations, the Bracken School Readiness Assessment (BSRA, [Pearson]) and the Movement Assessment Battery for Children (MABC, [Pearson]), were also administered to each child in Spanish. The BSRA assesses children’s knowledge of early preschool concepts such as letters, colors, and numbers, and requires them to respond verbally or by pointing to the correct answer among a group of options [15,16]. The MABC is a test of fine and gross motor skills and includes three domains: manual dexterity, aiming and catching, and balance [17]. The Test de Vocabulario en Imágenes Peabody (TVIP, [Pearson]), the Spanish adaptation of the Peabody Picture Vocabulary Test, was completed only by the cases since controls were evaluated between peaks of the COVID-19 pandemic (Figure 1) and researchers sought to limit contact time for control study visits. The TVIP assesses receptive vocabulary in Spanish and, similarly to the BSRA, requires children to select the correct image as stated by the examiner among a series of four images [18]. The TVIP is a commonly used neurodevelopmental assessment tool in Latin America [19]; composite Hispanic norms derived from testing conducted in Mexico and Puerto Rico were used for scoring the TVIP [18]. Head circumference, height, and weight were measured for each child, and BMI was calculated. Age-based percentiles for height and weight were determined by CDC growth charts [20], and those for head circumference were calculated using published national growth charts for older-age head circumference [21].

### 2.4. Statistical Analysis

Raw data were transferred from Colombia to the US-based team using a secure Dropbox portal. Data were entered into REDCap for storage and analysis [22]. Corresponding domains on BRIEF (Inhibit, Shift, Emotional Control, Working Memory, Plan/Organize, and General Executive Composite [GEC]) were compared between cohorts. The following pairs of categories on BRIEF-P/BRIEF-2 were considered analogous and thus compared between cohorts using T-scores from the BRIEF-P and BRIEF-2 manuals: Inhibitory Self-Control Index (ISCI) and Behavioral Regulation Index (BRI), Flexibility Index (FI) and Emotional Regulation Index (ERI), and Emotional Metacognition Index (EMI) and Cognitive Regulation Index (CRI) [23,24]. Because only the subscales which are included in both versions of the questionnaire were analyzed, several categories on the BRIEF-2 (which is longer than the BRIEF-P) were not analyzed individually, although they still factored into the three indices. On the BRIEF-2, these include Self-Monitor (sub-scale of the BRI), Initiate (sub-scale of the CRI), Task-Monitor (sub-scale of the CRI), and Organization of Materials (subscale of the CRI) [23]. For the BRIEF-P, all sub-scales were analyzed [24]. 

The standard scores on the MABC were compared rather than the raw scores, as the magnitude of the raw scores is not directly related to a child’s performance on each activity (e.g., 15 steps versus 20 for the line walking activity reflects size of steps, and not accuracy of balance). The raw scores on the BSRA were compared as a percentage of total correct items.

We also compared neurodevelopmental outcome scores within ZIKV-exposed cases between those with normal postnatal neuroimaging and those with non-specific imaging findings. We compared mean BRIEF T-scores overall, as well as in all sub-scales and in the BRI, ERI, and CRI. We also compared the mean MABC scores, the PEDI-CAT mean scaled scores, and the mean BSRA percent mastery.

We did not include *p*-values when reporting demographic, socioeconomic, and medical characteristics of the two cohorts because our sampling was not random with regard to these characteristics. Mann–Whitney U Tests were performed to compare scores between cases and controls as well as between cases with normal and non-specific postnatal neuroimaging. *p*-values were adjusted using the Benjamini–Hochberg false discovery rate (FDR) (significance, *p* < 0.1).

## 3. Results

### 3.1. Demographics

Demographic information for the 51 ZIKV-exposed cases and the 70 unexposed control participants is reported in Table 1. Cases were evaluated at a mean (SD) age of 5.3 (0.4) years and controls were evaluated at a mean age of 5.2 (0.3) years. Participant sex was comparable between cases and controls. Parent education was also similar between the two groups, with the majority of mothers and fathers reporting some or all of secondary school as their highest level of education completed. All cases and controls fall in the lowest Colombian socioeconomic stratum, which is a classification system based on characteristics of residential housing such as topography, type of road, public service availability, and house characteristics [25].

At the time of evaluation, a greater number of parents of ZIKV-exposed children reported a current concern about their child’s health compared to parents of control children (*p* = 0.001) (Table 1). A higher percentage of parents of cases also reported that their child had ever been diagnosed with an illness (Table 1). Illnesses reported among cases included an ear infection (n = 1), asthma (n = 2), adenitis (n = 1), allergies (n = 1), and malnutrition (n = 1). One control child had a reported diagnosis of asthma. No children qualitatively had microcephaly. Head circumference data collected during the 4–5 year study visits were inconsistent with data from surrounding timepoints; while we felt this could be explained by the hairstyles worn by the participants, we ultimately did not feel it was reliable and thus it is not reported. There were no differences in vision, hearing, or growth problems between the two groups nor were there any differences in the rates of children seeing a specialist doctor, or receiving physical, occupational, or speech therapy (Table 1). Parents of ZIKV-exposed children were more likely to report that their child had a behavior problem (*p* = 0.019) or mood problem (*p* = 0.010) than parents of controls (Table 1).

### 3.2. Neurodevelopmental Outcomes

There were differences in neurodevelopmental outcomes between the ZIKV-exposed cases and unexposed controls (Table 2). BRIEF scores for Shift and Emotional Control were higher in the cases than in the controls (FDR *p*-value = 0.056), leading to higher case emotional regulation index scores (FDR *p*-value = 0.020) and indicating lower function in emotional regulation skills. However, zero participants scored in the clinically significant range for the general executive composite. The mean MABC scores were similar in cases and controls. The mean TVIP scores for cases followed a normal distribution and were average relative to composite Hispanic norms (mean = 100, SD = 15) [18]. Across all BSRA sections except colors, ZIKV-exposed cases outperformed controls (FDR *p*-value < 0.001). The PEDI-CAT showed lower Mobility scores in cases compared to controls (FDR *p*-value < 0.001). However, Daily Activity scores were significantly higher in cases (FDR *p*-value = 0.004), and Responsibility scores trended higher in cases (FDR *p*-value = 0.102).

### 3.3. Zika Stigmatization and Maternal Mental Health

Mothers of the 51 ZIKV-exposed cases were surveyed during the study visits about the effect of their ZIKV diagnosis on their psychological state during and after pregnancy. A total of 12 mothers (23.5%) reported that they felt different from other mothers due to their ZIKV diagnosis during pregnancy. When asked to elaborate, the most common experience following ZIKV diagnosis during pregnancy was sadness (n = 7, 13.7%), followed by changes in relationships with partner/spouse (n = 3, 5.9%), anxiety (n = 1, 2%), depression (n = 1, 2%), and low self-esteem (n = 1, 2%). Zero mothers reported experiencing shame, isolation, change in friendships, or changes in sense of community because of their ZIKV diagnosis. No mothers reported that concerns about ZIKV affected their interactions with their child; however, 20 (39.2%) mothers reported being more likely to seek developmental follow-up for their child due to early concerns about ZIKV exposure.

### 3.4. Associations between Neurodevelopmental Outcomes and Postnatal Neuroimaging Findings

In total, 40 cases had both postnatal neuroimaging (head ultrasound and/or brain magnetic resonance imaging) during early infancy and evaluation at 4–5 years of age. A total of 17 out of 40 (42.5%), had mild, non-specific postnatal imaging findings and 23 had normal neuroimaging [9]. Included in the category “mild non-specific findings” are choroid plexus cysts, germinal matrix cysts, and a small frontal cyst (Supplementary Table S1). Also included were findings of minimal blood in the choroid plexus in one case, punctate white matter lesions, and lenticulostriate vasculopathy (Supplementary Table S1). Some cases had more than one of these findings (Supplementary Table S1). When neurodevelopmental scores were compared between the non-specific findings group and the normal imaging group, there were no differences in outcomes across the domains of executive function, motor ability, PEDI-CAT Daily Activity, or BSRA school readiness (Supplementary Table S2).

## 4. Discussion

This study presents the results of a neurodevelopmental evaluation of ZIKV-exposed children aged 4–5 years without CZS, and similarly aged unexposed control children in rural and semi-rural Colombia. Overall, children with ZIKV exposure who do not have CZS in Colombia appear to have a comparable motor developmental trajectory as their community peers by MABC evaluation but may have lower adaptive mobility skills as measured by the PEDI-CAT. Performance on the TVIP and BSRA yielded age-appropriate results for ZIKV-exposed cases. Mean BRIEF scores were also in the normal range; however, there were differences in BRIEF scores between cases and controls in the Shift and Emotional Control categories. This finding is consistent with BRIEF scores in the cases compared to the controls, when previously analyzed at age 3–4 years [7]. The consistent finding of increased elevation in this category of testing among cases at both time points indicates that executive function skills may be vulnerable to the long-term impacts of antenatal ZIKV exposure. This observation is further supported by the higher proportion of parents of ZIKV-exposed cases reporting behavior and mood problems in their children compared to parents of unexposed controls. Thus, this study highlights the need for the continued follow-up of emotional regulation in children with antenatal ZIKV exposure to older ages.

To our knowledge, no other studies of normocephalic children with antenatal ZIKV-exposure have specifically noted differences in the emotional regulation domain. However, both animal and human studies strongly suggest that pre and perinatal insults pose risks to the development of executive function [26,27,28,29], supporting the possibility of a link between congenital ZIKV infection and changes in emerging emotional or behavioral regulatory ability. Notably, all data which point to potential differences in cases’ emotional regulation are from caregiver report measures. Caregivers of ZIKV-exposed cases expressed higher rates of concern about the health of their children, and many reported an inclination to seek developmental follow-up due to their ZIKV diagnosis. It is possible that parents or caregivers of children with ZIKV exposure may be more sensitive to alterations in their child’s behavior due to their concern over the effects of ZIKV exposure and, thus, could be more likely to report concerns in behavioral or emotional domains than parents or caregivers of unexposed controls. In the future, it would be valuable to also examine these domains using child-based observational measures to eliminate this potential bias. Higher parental anxiety has also been found to be associated with behavioral and emotional dysregulation in young children [30,31]. It would be useful to study the connection between parental mental health following ZIKV diagnosis and subsequent child development in greater detail to better elucidate the potential effects of stigmatization and anxiety following a ZIKV diagnosis on child development.

Although scores on the PEDI-CAT Mobility domain were significantly lower in ZIKV-exposed cases compared to controls, scores on the MABC did not differ between groups. PEDI-CAT Mobility scores come from adaptive motor functions in the home and are reported by parent or caregiver responses, so it may be that ZIKV-exposed children have a mild difference in adaptive motor functions which is not detected by the MABC. Thus, it remains unclear whether motor ability is impacted by ZIKV exposure. Researchers have found that confirmed congenital Zika infection at birth does correlate with delays in motor acquisition up to an age of three years [32], and ZIKV exposure may also lead to higher rates of motor delay [6,33]. However, other studies have not seen a difference in motor outcomes among children with ZIKV exposure up to an age of four years [34,35,36]. A continued longitudinal follow-up is warranted to determine whether differences in motor abilities will emerge as ZIKV-exposed children get older.

The examination of these neurodevelopmental outcomes in the context of early postnatal imaging findings enhances this study by ruling out one potential driver of differences between cases and controls. In an earlier analysis of the relationship between imaging findings and neurodevelopment in this cohort, we highlighted a trend towards lower motor developmental skills as measured by the Alberta Infant Motor Scales in cases with non-specific postnatal imaging findings [8]. However, in this study by the age of school entry, any early delays in motor ability related to imaging findings appear to have been ameliorated. The imaging findings in these children were all mild, and felt to be non-specific, in that they can be seen in a variety of types of patients and are not specific to in utero ZIKV exposure. It is not known whether these findings occurred *because* of antenatal ZIKV exposure, or would have been present regardless, but the higher frequency of seeing them in this group of children is felt to indicate that they are likely related. Nevertheless, the presence of these specific neuroimaging findings in ZIKV-exposed children without CZS does not appear to indicate any increased risk on neurodevelopment at an age of 4–5 years.

A challenge of this study was managing the confounding influence of the COVID-19 pandemic, which posed serious social and educational restraints on children in Colombia. The unexposed control children were screened by birth date to preclude asymptomatic ZIKV exposure during the first half of gestation and thus were an average of 1 year and 4 months older than the 51 ZIKV-exposed cases. The control children had their 4–5-year-old evaluations between January and February 2021, a period which coincided with continued and prolonged school closures due to the COVID-19 pandemic in Colombia (Figure 1). The children evaluated should have been in the Colombian equivalent of kindergarten but had not been able to start school at that time. Options for synchronous virtual schooling were limited for most children in our study, with parents commonly reporting that school was being conducted via television, WhatsApp, or not at all [37]. The quarantine periods in Colombia were prolonged in this region, and children were likely fairly isolated from other environments outside of their home and from other children or adults [37]. The ZIKV-exposed cases, on the other hand, are younger and were able to avoid the pandemic-imposed delays to schooling (Figure 1), beginning kindergarten at the typical age of five years. These circumstances likely explain why cases scored higher than controls on the BSRA and the Daily Activities domain of the PEDI-CAT.

The COVID-19 pandemic also limited the study team’s ability to complete the full ZIKV Outcome Toolbox for the controls. Due to efforts to shorten the length of the study visits and to minimize potential exposures to COVID-19, no TVIP data were collected for controls during the visits, which limits our ability to contextualize the TVIP scores of the ZIKV-exposed cases at an age of 4–5 years. Therefore, we are unable to compare the scores on the TVIP from the cases to those of typically developing children from the same geographic, socioeconomic, and cultural context, and thus relied on normative data from Mexico and Puerto Rico to determine whether the scores in our cases represented age-appropriate development in this population [18]. However, other studies have found delays in the language domain among children with ZIKV-exposure during the intra-uterine period [33,38,39]. Thus, it will be important to enhance our study of language development at future timepoints compared to an unexposed control cohort.

## 5. Conclusions

Although the COVID-19 pandemic presented a challenge to early childhood research, children with in utero ZIKV exposure appear to have an overall positive developmental trajectory but may experience risks to neurodevelopment in childhood in areas of emotional regulation and adaptive mobility. There is a need for the continued follow-up of children with prenatal ZIKV exposure to discover its long-term effects.

## Figures and Tables

**Table 1:** Demographics and medical history of ZIKV-exposed and unexposed children.

	ZIKV-Exposed Cases (N = 51)	Unexposed Controls (N = 70)
**Mean (SD) Age at Visit (years)**	5.3 (0.4)	5.2 (0.3)
Range of Ages at Visit (years)	4.6–5.8	4.9–5.9
**Male Sex at Birth**	21 (41.2%)	33 (47.1%)
**Mean BMI (SD)**	14.8 (1.6)	15.1 (1.3)
**Maternal Education**		
Primary School	5 (9.8%)	9 (12.9%)
Secondary School	36 (70.6%)	48 (68.6%)
Technical Institute	7 (13.7%)	10 (14.3%)
University	0 (0%)	0 (0%)
None	1 (2.0%)	2 (2.9%)
NR	2 (3.9%)	1 (1.4%)
**Paternal Education**		
Primary School	7 (13.7%)	16 (22.9%)
Secondary School	35 (68.6%)	43 (61.4%)
Technical Institute	3 (5.9%)	3 (4.3%)
University	0 (0%)	3 (4.3%)
None	2 (3.9%)	1 (1.4%)
NR	4 (7.8%)	4 (5.7%)
**Parents/Caregivers Worried About the Health of Their Child**	17 (33.3%)	5 (7.1%)
**Vision Problems**	1 (2.0%)	0 (0%)
**Hearing Problems**	0 (0%)	2 (2.9%)
**Growth Problems**	1 (2.0%)	0 (0%)
**Behavior Problems**	8 (15.7%)	2 (2.9%)
**Mood Problems**	7 (13.7%)	1 (1.4%)
**Diagnosed with an Illness**	7 (13.7%)	1 (1.4%)
**Sees Specialist Doctor**	1 (%)	2 (2.9%)
**Therapy** (physical, occupational, or speech)	1 (2.0%)	1 (1.4%)

SD: standard deviation; BMI: body mass index; NR: no response.

**Table 2:** Neurodevelopmental performance in both ZIKV-exposed and unexposed children.

Assessment	ZIKV-Exposed Cases (N = 51)	Unexposed Controls (N = 70)	*p*-Value, FDR
**BRIEF T-Score: Mean (SD)**			
Inhibit	51.6 (8.9)	56.0 (12.0)	0.169
Emotional Control	51.9 (7.9)	49.2 (13.0)	0.056 *
Shift	53.8 (9.5)	50.2 (10.7)	0.056 *
Working Memory	49.0 (9.1)	55.0 (14.6)	0.204
Plan and Organize	48.4 (6.4)	52.3 (14.5)	0.612
Behavioral Regulation Index (ISCI/BRI)	51.4 (8.1)	53.6 (13.6)	0.920
Emotional Regulation Index (FI/ERI)	53.5 (9.1)	49.7 (13.5)	0.020 **
Cognitive Regulation Index (EMI/CRI)	48.9 (7.5)	53.2 (13.3)	0.321
General Executive Composite	51.0 (7.9)	53.6 (15.3)	0.948
**MABC Standard Score: Mean (SD)**			
Manual Dexterity	25.3 (6.9)	24.3 (6.5)	0.653
Aiming and Catching	22.4 (5.4)	20.7 (4.8)	0.216
Balance	31.6 (6.7)	30.2 (7.8)	0.612
Overall	79.8 (13.5)	75.0 (14.8)	0.204
**TVIP Raw Score: Mean (SD)**	95.7 (19.0)	-	-
**PEDI-CAT Scaled Score: Mean (SD)**			
Mobility	67.3 (4.2)	75.5 (1.4)	<0.001 **
Daily Activity	58.2 (5.4)	54.8 (1.9)	0.004 **
Responsibility	46.8 (13.7)	44.4 (11.0)	0.102
Social/Cognitive	63.6 (4.0)	62.6 (5.7)	0.368
**BSRA % Mastery: Mean (SD)**			
Colors	75.4 (32.8)	62.2 (40.6)	0.120
Letters	30.2 (19.4)	9.93 (16.1)	<0.001 **
Numbers	45.1 (27.3)	26.3 (30.3)	<0.001 **
Sizes and Comparisons	55.2 (10.9)	33.7 (17.2)	<0.001 **
Shapes	48.6 (15.9)	29.5 (15.3)	<0.001 **
Total	49.6 (15.4)	30.8 (16.2)	<0.001 **

BRIEF: Behavior Rating Inventory of Executive Function; BSRA: Bracken School Readiness Assessment; FDR: false discovery rate; SD: standard deviation; MABC: Movement Assessment Battery for Children; PEDI-CAT: Pediatric Evaluation of Disability Inventory—Computer Adaptive Test; TVIP: Test de Vocabulario en Imágenes Peabody (Peabody Picture Vocabulary Test). *p*-values adjusted for comparisons using the FDR, * Indicates a *p*-value less than 0.1, ** indicates a *p*-value less than 0.05.

## Data Availability

The data presented in this study are available upon reasonable request from the corresponding author. The data are not publicly available due to privacy considerations for participating children and families.

## Supplementary Materials

The following supporting information can be downloaded at: https://www.mdpi.com/article/10.3390/pathogens13020170/s1, Table S1: Non-specific imaging findings from infant head ultrasound or brain MRI in ZIKV-exposed cases (N = 17); Table S2: Neurodevelopmental outcomes at an age of 4–5 years in ZIKV-exposed cases with and without infant ultrasound findings.

## References

[1] Gubler D.J., Vasilakis N., Musso D. (2017). History and Emergence of Zika Virus. J. Infect. Dis..

[2] Ikejezie J., Shapiro C.N., Kim J., Chiu M., Almiron M., Ugarte C., Espinal M.A., Aldighieri S. (2017). Zika Virus Transmission—Region of the Americas. MMWR Morb. Mortal. Wkly. Rep..

[3] Pacheco O., Beltrán M., Nelson C.A., Valencia D., Tolosa N., Farr S.L., Padilla A.V., Tong V.T., Cuevas E.L., Espinosa-Bode A. (2020). Zika Virus Disease in Colombia—Preliminary Report. N. Engl. J. Med..

[4] Walker C.L., Little M.-T.E., Roby J.A., Armistead B., Gale M., Rajagopal L., Nelson B.R., Ehinger N., Mason B., Nayeri U. (2019). Zika Virus and the Nonmicrocephalic Fetus: Why We Should Still Worry. Am. J. Obstet. Gynecol..

[5] Adebanjo T., Godfred-Cato S., Viens L., Fischer M., Staples J.E., Kuhnert-Tallman W., Walke H., Oduyebo T., Polen K., Peacock G. (2017). Update: Interim Guidance for the Diagnosis, Evaluation, and Management of Infants with Possible Congenital Zika Virus Infection—United States, October 2017. MMWR Morb. Mortal. Wkly. Rep..

[6] Marbán-Castro E., Vazquez Guillamet L.J., Pantoja P.E., Casellas A., Maxwell L., Mulkey S.B., Menéndez C., Bardají A. (2022). Neurodevelopment in Normocephalic Children Exposed to Zika Virus in Utero with No Observable Defects at Birth: A Systematic Review with Meta-Analysis. Int. J. Environ. Res. Public Health.

[7] Mulkey S.B., Peyton C., Ansusinha E., Corn E., Arroyave-Wessel M., Zhang A., Biddle C., Gutierrez C., Sorkar A., Cure A. (2023). Preschool Neurodevelopment in Zika Virus-Exposed Children without Congenital Zika Syndrome. Pediatr. Res..

[8] Mulkey S.B., Arroyave-Wessel M., Peyton C., Bulas D.I., Fourzali Y., Jiang J., Russo S., McCarter R., Msall M.E., du Plessis A.J. (2020). Neurodevelopmental Abnormalities in Children with In Utero Zika Virus Exposure without Congenital Zika Syndrome. JAMA Pediatr..

[9] Mulkey S.B., Bulas D.I., Vezina G., Fourzali Y., Morales A., Arroyave-Wessel M., Swisher C.B., Cristante C., Russo S.M., Encinales L. (2019). Sequential Neuroimaging of the Fetus and Newborn with In Utero Zika Virus Exposure. JAMA Pediatr..

[10] Instituto Nacional de Salud Casos Positivos de COVID-19 en Colombia 2020. https://www.ins.gov.co/Noticias/Paginas/Coronavirus.aspx.

[11] García Fernández T., González-Pienda J.A., Rodríguez Pérez C., Álvarez García D., Álvarez Pérez L. (2014). Psychometric Characteristics of the BRIEF Scale for the Assessment of Executive Functions in Spanish Clinical Population. Psicothema.

[12] Bausela Herreras E. (2019). BRIEF-P: Validation Study in Children in Early Childhood with Neurodevelopmental Disorders. SAGE Open.

[13] Hendrickson N.K., McCrimmon A.W. (2019). Test Review: Behavior Rating Inventory of Executive Function®, Second Edition (BRIEF®2) by Gioia, G.A., Isquith, P.K., Guy, S.C., & Kenworthy, L. Can. J. Sch. Psychol..

[14] Haley S.M., Coster W.J., Dumas H.M., Fragala-Pinkham M.A., Kramer J., Ni P., Tian F., Kao Y.-C., Moed R., Ludlow L.H. (2011). Accuracy and Precision of the Pediatric Evaluation of Disability Inventory Computer-Adaptive Tests (PEDI-CAT). Dev. Med. Child Neurol..

[15] Bracken B.A. (2007). Bracken School Readiness Assessment.

[16] Ortiz A., Clinton A., Schaefer B.A. (2015). Construct Validity Evidence for Bracken School Readiness Assessment, Third Edition, Spanish Form Scores. Psychol. Sch..

[17] Henderson S.E., Sugden D., Barnett A.L. (1992). Movement Assessment Battery for Children-2. Res. Dev. Disabil..

[18] Dunn L., Lugo D., Padilla E., Dunn L. (1986). Test de Vocabulario En Imagines Peabody.

[19] Williams M.E., Corn E.A., Ransanz S.M., Berl M.M., Andringa-Seed R., Mulkey S.B. Neurodevelopmental Assessments Used to Measure Preschoolers’ Cognitive Development in Latin America: A Systematic Review. J. Pediatr. Psychol..

[20] Kuczmarski R.J., Ogden C.L., Grummer-Strawn L.M., Flegal K.M., Guo S.S., Wei R., Mei Z., Curtin L.R., Roche A.F., Johnson C.L. (2000). CDC Growth Charts: United States. Adv Data.

[21] Nellhaus G. (1968). Head Circumference from Birth to Eighteen Years. Practical Composite International and Interracial Graphs. Pediatrics.

[22] Harris P.A., Taylor R., Thielke R., Payne J., Gonzalez N., Conde J.G. (2009). Research Electronic Data Capture (REDCap)—A Metadata-Driven Methodology and Workflow Process for Providing Translational Research Informatics Support. J. Biomed. Inf..

[23] Gioia G.A., Isquith P.K., Guy S.C., Kenworthy L. (2000). Behavior Rating Inventory of Executive Function: BRIEF.

[24] Gioia G.A., Andrwes K., Isquith P.K. (1996). Behavior Rating Inventory of Executive Function-Preschool Version (BRIEF-P).

[25] Chica-Olmo J., Sánchez A., Sepúlveda-Murillo F.H. (2020). Assessing Colombia’s Policy of Socio-Economic Stratification: An Intra-City Study of Self-Reported Quality of Life. Cities.

[26] Brown A.S., Vinogradov S., Kremen W.S., Poole J.H., Deicken R.F., Penner J.D., McKeague I.W., Kochetkova A., Kern D., Schaefer C.A. (2009). Prenatal Exposure to Maternal Infection and Executive Dysfunction in Adult Schizophrenia. Am. J. Psychiatry.

[27] Hostinar C.E., Stellern S.A., Schaefer C., Carlson S.M., Gunnar M.R. (2012). Associations between Early Life Adversity and Executive Function in Children Adopted Internationally from Orphanages. Proc. Natl. Acad. Sci. USA.

[28] Teigset C.M., Mohn C., Rund B.R. (2020). Perinatal Complications and Executive Dysfunction in Early-Onset Schizophrenia. BMC Psychiatry.

[29] Hunter S.J., Schuh J.M., Hunter S.J., Sparrow E.P. (2012). Executive Functions after Congenital and Prenatal Insults. Executive Function and Dysfunction: Identification, Assessment and Treatment.

[30] Fields A., Harmon C., Lee Z., Louie J.Y., Tottenham N. (2021). Parent’s Anxiety Links Household Stress and Young Children’s Behavioral Dysregulation. Dev. Psychobiol..

[31] Sweeney S., Wilson C. (2023). Parental Anxiety and Offspring Development: A Systematic Review. J. Affect. Disord..

[32] Hcini N., Kugbe Y., Rafalimanana Z.H.L., Lambert V., Mathieu M., Carles G., Baud D., Panchaud A., Pomar L. (2021). Association between Confirmed Congenital Zika Infection at Birth and Outcomes up to 3 Years of Life. Nat. Commun..

[33] Blackmon K., Evans R., Fernandes M., Landon B., Noel T., Macpherson C., Cudjoe N., Burgen K.S., Punch B., Krystosik A. (2022). Neurodevelopment in Normocephalic Children with and without Prenatal Zika Virus Exposure. Arch. Dis. Child..

[34] Grant R., Fléchelles O., Tressières B., Dialo M., Elenga N., Mediamolle N., Mallard A., Hebert J.-C., Lachaume N., Couchy E. (2021). In Utero Zika Virus Exposure and Neurodevelopment at 24 Months in Toddlers Normocephalic at Birth: A Cohort Study. BMC Med..

[35] Fernandes M., Evans R., Cheng M., Landon B., Noël T., Macpherson C., Cudjoe N., Burgen K.S., Waechter R., LaBeaud A.D. (2023). Does Intra-Uterine Exposure to the Zika Virus Increase Risks of Cognitive Delay at Preschool Ages? Findings from a Zika-Exposed Cohort from Grenada, West Indies. Viruses.

[36] Alger J., Cafferata M.L., López R., Wiggins L.D., Callejas A., Castillo M., Fúnes J., Rico F., Valencia D., Varela D. (2023). Neurodevelopmental Assessment of Normocephalic Children Born to Zika Virus Exposed and Unexposed Pregnant People. Pediatr. Res..

[37] Williams M.E., Berl M.M., Corn E., Ansusinha E., Arroyave-Wessel M., Zhang A., Cure C., Mulkey S.B. (2023). Positive and Negative Effects of the COVID-19 Pandemic on Families of Young Children in Rural Colombia and Implications for Child Outcome Research. Child Care Health Dev..

[38] Peçanha P.M., Gomes Junior S.C., Pone S.M., Pone M.V., Vasconcelos Z., Zin A., Vilibor R.H.H., Costa R.P., Meio M.D.B.B., Nielsen-Saines K. (2020). Neurodevelopment of Children Exposed Intra-Uterus by Zika Virus: A Case Series. PLoS ONE.

[39] Nielsen-Saines K., Brasil P., Kerin T., Vasconcelos Z., Gabaglia C.R., Damasceno L., Pone M., Abreu de Carvalho L.M., Pone S.M., Zin A.A. (2019). Delayed Childhood Neurodevelopment and Neurosensory Alterations in the Second Year of Life in a Prospective Cohort of ZIKV-Exposed Children. Nat. Med..

