# Supplementary material for: Neurodevelopmental Outcomes of Normocephalic Colombian Children with Antenatal Zika Virus Exposure at School Entry
Source: Pathogens. 2024 Feb 13;13(2):170. doi: 10.3390/pathogens13020170 (PMC10892822; doi:10.3390/pathogens13020170)
Supplement: Supplementary file 1 [file pathogens-13-00170-s001.zip › pathogens-2812619-supplementary.pdf]

Supplementary Table S1: Nonspecific imaging findings from infant head ultrasound or brain MRI in ZIKV-exposed cases (N=17).

Child	Type of Imaging	Finding
**1**	US	CPC (U)
**2**	US	GM cyst (U)
**3**	US	GM cysts (B)
**4**	US	GM cysts (B)
**5**	US/MRI	GM cyst (U)
**6**	MRI	Minimal blood products in choroid plexus
**7**	MRI	Punctate white matter hemorrhage (U)
**8**	US	LSV
**9**	US	LSV
**10**	US	CPC (U); LSV
**11**	US	CPC (U)
**12**	US	Small frontal cyst (U)
**13**	US	CPC (U); LSV
**14**	MRI	Punctate lesion in parietal white matter (U)
**15**	US	CPC (U)
**16**	US	CPC, GM cyst (U)
**17**	US	GM cyst (U)

US: ultrasound, MRI: magnetic resonance imaging, U: unilateral finding, B: Bilateral finding, CPC: choroid plexus cyst, LSV: lenticulostriate vasculopathy, GM: germinal matrix

Supplementary Table S2: Neurodevelopmental outcomes at age 4-5 years in ZIKV-exposed cases with and without infant mild neuroimaging findings.

	Mild nonspecific imaging findings (N=17)	Normal imaging (N=23)	p-value, FDR
**BRIEF T-Score: Mean (SD)**			
Inhibit	51.6 (9.8)	51.0 (9.1)	0.843
Emotional Control	51.1 (8.8)	52.3 (8.4)	0.843
Shift	52.6 (10.0)	53.5 (10.0)	0.843
Working Memory	50.2 (8.2)	47.1 (10.3)	0.843
Plan and Organize	47.8 (5.8)	47.7 (6.8)	0.843
Behavioral Regulation Index (ISCI/BRI)	51.2 (8.6)	50.6 (8.4)	0.843
Emotional Regulation Index (FI/ERI)	51.8 (9.6)	54.3 (10.5)	0.843
Cognitive Regulation Index (EMI/CRI)	49.1 (7.4)	47.6 (7.5)	0.843
General Executive Composite	50.8 (8.6)	49.9 (7.9)	0.843
**MABC Standard Score: Mean (SD)**			
Manual Dexterity	25.4 (7.8)	26.4 (6.0)	0.843
Aiming and Catching	23.8 (4.3)	22.4 (5.9)	0.843
Balance	32.8 (5.4)	31.7 (5.2)	0.843
Overall	83.4 (9.7)	80.7 (11.4)	0.843
**PEDI-CAT Scaled Score: Mean (SD)**			
Mobility	66.9 (4.0)	68.0 (4.7)	0.843
Daily Activity	57.9 (5.6)	59.0 (5.7)	1
Responsibility	43.5 (17.1)	48.7 (11.9)	1
Social/Cognitive	63.3 (3.8)	64.0 (4.8)	1
**BSRA % Mastery: Mean (SD)**			
Colors	70.6 (37.0)	79.5 (29.0)	1
Letters	34.6 (24.3)	31.9 (15.9)	1
Numbers	40.6 (28.2)	50.9 (25.3)	1
Sizes and Comparisons	55.2 (11.0)	59.0 (8.8)	1
Shapes	48.4 (18.5)	48.4 (16.6)	1
Total	48.7 (18.1)	52.8 (13.4)	1

BRIEF: Behavior Rating Inventory of Executive Function; BSRA: Bracken School Readiness Assessment; FDR: false discovery rate; SD: standard deviation; Movement ABC: Movement Assessment Battery for Children; PEDI-CAT: Pediatric Evaluation of Disability Inventory – Computer Adaptive Test. P-values adjusted for comparisons using the FDR.
